# Kaolin particle films disrupt landing, settling behavior and feeding of *Trioza erytrae* on lemon plants

**DOI:** 10.1002/ps.7095

**Published:** 2022-08-11

**Authors:** Diogo Félix Oliveira, Jacinto Benhadi‐Marín, Joana Neto, Lorena Sanz, Elisa Garzo, Ana Aguiar, Alberto Fereres, José Alberto Pereira

**Affiliations:** ^1^ Centro de Investigação de Montanha (CIMO), Instituto Politécnico de Bragança Campus Sta Apolónia Bragança Portugal; ^2^ Laboratório Associado para a Sustentabilidade e Tecnologia em Regiões de Montanha, Instituto Politécnico de Bragança Campus Sta Apolónia Bragança Portugal; ^3^ GreenUPorto—Sustainable Agrifood Production Research Centre/Inov4Agro, DGAOT Faculty of Sciences of the University of Porto Vairão Portugal; ^4^ Instituto de Ciencias Agrarias, Consejo Superior de Investigaciones Científicas, ICA‐CSIC Madrid Spain

**Keywords:** huanglongbing, colonization, spray, probing, electrical penetration graphs

## Abstract

**BACKGROUND:**

The citrus greening disease or Huanglongbing (HLB) is the most devastating disease of citrus crops. *Trioza erytreae* is a vector of HLB. Since its introduction in Europe, the insect reached the northern region of Spain and the southern region of Portugal, threatening relevant citrus production areas. Limiting the spread of HLB vectors is mandatory to prevent this disease. In this work, we assessed the effect of kaolin, a white mineral clay, on the landing, settling behavior and feeding behavior of *Trioza erytreae* on lemon plants.

**RESULTS:**

After kaolin application, the number of plants on which the insect was found was significantly lower than on untreated plants in the laboratory and in the field. Moreover, there were significantly fewer *T. erytreae* and a shorter duration of phloem‐related events on kaolin‐treated than untreated plants.

**CONCLUSION:**

The use of kaolin could be a suitable and efficient tool for inclusion into integrated pest management programs or organic production to reduce populations of *T. erytreae* and subsequently limit the spread of HLB in citrus crops. © 2022 The Authors. *Pest Management Science* published by John Wiley & Sons Ltd on behalf of Society of Chemical Industry.

## Graphical abstract

White kaolin significantly reduced the number of *Trioza erytreae* adults settled on lemon plants compared with control plants. Moreover, the psyllid fed significantly less on phloem in kaolin‐treated plants.

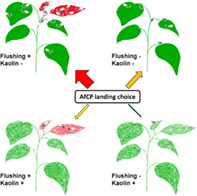

## 1 INTRODUCTION

*Trioza erytreae* del Guercio is an important threat to citriculture in the Mediterranean region. In the last 7 years, the pest has been expanding from the northwestern region of the Iberian Peninsula to southern Portugal and the Cantabric region of Spain. This psyllid has recently reached the Algarve region of Portugal (DGAV, 2016–2021 http://www.dgv.minagricultura.pt/portal/page/portal/DGV/genericos?generico=221911&cboui=221911)), an area where citrus crops are widely grown. Citrus greening disease or Huanglongbing (HLB) is a devastating disease considered the most important one of citrus crops of which *T. erytreae* is one of the two vectors. Therefore, vector management could be the most important strategy to control HLB.^1^ Among the different control options and cultural tactics, trap crops^2^ or reflective mulches^3^ were proven to reduce the spread of HLB in citrus orchards grown in Brazil and USA. Furthermore, studies developed with *Diaphorina citri* Kuwayama, the other HLB vector, demonstrated that using particle film technologies such as kaolin clay applied on citrus plants can reduce the host‐finding ability and interfere with landing and settling behavior, thus preventing the transmission and spread of the disease in young orange plantations.^4^

Particle film technologies encompass aqueous formulations made from chemically inert clay or mineral particles formulated explicitly for coating. These clays have been used widely to reduce the damage caused by insects, diseases and solar injury.^5^ Kaolin is an aluminosilicate mineral clay, chemically inert over a wide pH range. White kaolin has been used to protect against pests in a wide range of crops such as apple, almond, cabbage, cotton, olive, pear, pecan, tomatoes, walnut and wine grape, and is proven to be very effective against psyllids.^6^, ^7^, ^8^, ^9^, ^10^, ^11^, ^12^, ^13^, ^14^, ^15^, ^16^, ^17^ A pool of studies reported kaolin as an effective substance in reducing the populations of pests from orders Coleoptera, Diptera, Hemiptera and Lepidoptera,^10^, ^11^, ^15^, ^16^, ^17^, ^18^, ^19^ acting as a repellent or barrier for pests and affecting the recognition and attractiveness of host plants.^20^ Regarding the citrus agroecosystem, the effect of kaolin on the behavior of *D. citri* populations has been tested on *Citrus sinensis* L. (sweet orange) in Florida (USA).^21^ Also, recent work conducted in open field orange crops in Brazil shows that the application of kaolin sprays at 7‐ and 14‐day intervals reduced psyllid densities by 80% compared to untreated trees.^22^

The electrical penetration graph (EPG) technique is a useful tool to study the feeding behavior of sap‐sucking insects.^23^, ^24^, ^25^ EPG also has been used to correlate psyllid‐feeding activities with the transmission of *Candidatus* Liberibacter^26^, ^27^, ^28^, ^29^ and to study the effect of systemic insecticides on the probing and feeding behavior of psyllids.^30^, ^31^ In addition, the EPG technique has been used to assess the host plant preference of *Bactericera cockerelli* (Šulc)^32^ on Solanceae and *T. erytreae* on citrus.^33^ In the present study, we used EPG to determine the effects of kaolin treatments in citrus on the probing and feeding behavior of *T. erytreae*.

A comprehensive review on cultural methods and other management strategies against *Diaphorina citri* and HLB has been published recently.^34^ However, information on the efficacy of kaolin particle film technology as a way to interfere with the host plant‐finding ability, settling behavior,‐ and probing of *T. erytreae* on citrus plants is lacking. Accordingly, the objectives of this work were: (i) to study the effect of the application of kaolin on the landing and settlement of adult individuals of *T. erytreae* on lemon plants using dual‐choice assays in the laboratory and open field mark‐release‐recapture experiments; and (ii) to assess the impact of kaolin particle films on the probing and feeding behavior of *T. erytreae* on lemon plants.

## 2 MATERIAL AND METHODS

### 2.1 Dual‐choice test in laboratory

#### 2.1.1 Insects origin and rearing

The adults of *T. erytreae* (10–15 days old) used for experiments were obtained from a healthy breeding population in lemon plants started in 2019 in the pedagogical orchard ‘Quinta do Crasto’ of the Agrarian Campus of Vairão (University of Porto) (41° 19′ 38.5″ N, 8° 40′ 32.4″ W). The rearing was conducted in an insectary maintained at 23 ± 5 °C, 70 ± 10% relative humidity (RH), light source 4500 K and 6000 K LED lamps (1200 lm) and a photoperiod of 14 h:10 h, light:dark until the assay started. The individuals were maintained in 1–2‐year‐old *Citrus limon* plants (cv. Eureka and Valencia). Plants were fertilized monthly with slow‐release complex granulated blue fertilizer NPK (S) 12‐8‐16. Also, fertigation was applied with Welgro Micromix solution. Corrections were made with Sequestrene–Iron Chelate (6.2%) when needed. Pruning of shoots was done regularly to boost growth to ensure the continuous availability of lemon flushes for egg‐laying and further development of nymphs.

#### 2.1.2 Dual‐choice test in the laboratory

The preference of adults of *T. erytreae* for control or kaolin‐treated plants was assessed using a dual choice test. For this purpose, 60 *C. limon* (cv. Eureka) seedlings were selected for laboratory assays. Each plant had five leaves, an average height of 30 cm, and presented new shoots in V2/V3 state at the time when the experiments started. All of the plants were watered and fertilized regularly.

The control encompassed 30 plants, whereas another 30 plants were sprayed with kaolin. Control plants were sprayed with distilled water. Treated plants were sprayed with a 3% (w/v) kaolin solution (Surround® WP). The kaolin solution was applied using a hand sprayer (Matabi Berry®1.5 L, Goizper Group, Gipuzkoa, Spain). Each plant was sprayed 72 h and 48 h before the assay. The leaves were sprayed until runoff on both the top and bottom surfaces.

The dual choice assay was conducted using 30 cages made of plastic and cloth to allow ventilation. A pair of lemon seedlings separated 20 cm from each other were placed inside each cage (i.e. one control plant and one kaolin‐treated plant) (Supporting information, Fig. S1). All cages were placed in a growth chamber (Fitoclima 600, Aralab®) at 25 °C, 14 h:10 h, light:dark photoperiod, 70 ± 5% RH, and direct light from lighting above to guarantee the same light intensity and direction for all cages.

A total of 16 adults of *T. erytreae* (1:1 male–female ratio) were released inside each cage on a circular platform equidistant from the two plants. Before release, insects were kept in the cold for 5 min at 5 °C to ease manipulation. After 1, 4, 8, 24, 48 and 72 h, the number of individuals settled on each plant and the number of dead individuals were recorded.

### 2.2 Effect of kaolin on the probing and feeding behavior of *T. erytreae*

The EPG technique was used to evaluate the probing and feeding behavior of *T. erytreae* on lemon (cv. Eureka) seedlings (at the four‐leaf stage) previously sprayed with kaolin (Surround® WP 3%). Plants sprayed with water were used as an untreated control. The methodology used was similar to that of Bonani *et al*.^26^ Plants were sprayed with kaolin or with water (untreated control) 24 h before starting experiments. Kaolin application was repeated twice until runoff to ensure a uniform layer of white film covering the leaves where EPG experiments were conducted.

The EPG recordings were obtained using a DC monitor, GIGA‐8d model (EPG Systems, Wageningen, the Netherlands),^24^ adjusted to ×100 gain. Young adult female psyllids were maintained in a refrigerator at 4 °C for 30 s to reduce their activity and then immediately immobilized by a vacuum device under a dissection microscope. Then, a gold wire (2 cm long × 18.5 μm diameter; Sigmund Cohn, Mount Vernon, NY, USA), previously connected to a copper electrode (3 cm long × 1 mm diameter), was attached to the psyllid prothorax using water‐based silver conductive paint glue (EPG Systems). The insects were connected to the EPG device and placed on the abaxial surface of the last expanded lemon leaf on control and treated kaolin plants. Another electrode (copper, 10 cm long × 2 mm wide) was inserted into the pot substrate containing the lemon seedling. The EPG recordings lasted 8 h and were conducted inside a Faraday cage (100 × 110 × 90 cm) to avoid electrical noise. Only those EPG recordings with an optimal signal quality were considered for analysis. The EPG recordings were acquired and analyzed using stylet+ software for Windows (EPG Systems). A total of 15 and 14 EPG recordings (8 h) (replicates) on control lemon plants (treated with water) and plants treated with kaolin were recorded and analyzed, respectively. Recordings in which psyllids exhibited an irregular behavior that deviates from what is considered standard [i.e. total duration of nonprobing (Np) >75%] were discarded. A single psyllid and plant combination were used for each replicate. The EPG waveforms associated with specific stylet tip positions and insect activities were characterized according to previous EPG studies with the psyllids *D. citri* (Bonani)^26^ and *B. trigonica*.^35^ The EPG waveforms considered were: Np, stylets not inserted into the leaf tissue; C, stylet intracellular penetration into the leaf tissue; D, phloem contact; E1, salivation into phloem sieve elements; E2, passive phloem sap ingestion; and G, active xylem sap ingestion.

In order to analyze the impact of kaolin treatment on probing and feeding behavior of *T. erytreae*, a selected set of EPG nonsequential variables (Table 1) were calculated, namely: the number of waveform events for each insect (the number of times that a waveform occurs for each insect); the total waveform duration for each insect (the sum of overall occurrences of a waveform for each insect); and the mean duration of waveform events for each insect (the total waveform duration divided by the number of waveform events for each insect). The following sequential variables were calculated: time to first probe from the start of EPG, time from the start of EPG to first sustained E2 (>10 min), and time to the 1st E2 from the start of 1st probe. If a particular waveform event did not exist, then the value was considered missing data.

**Table 1:** EPG variables of *Trioza erytreae* exposed to untreated and kaolin‐treated lemon plants during 8 h of recording

Variable type	Kaolin‐treated plants		Control plants		*P*
	PPW	Mean ± SE	PPW	Mean ± SE	
**Nonsequential variables**					
Np	14/14	4.93 ± 0.84^a^	15/15	11.87 ± 1.68^b^	0.003
Probe	14/14	4.86 ± 0.83^a^	15/15	11.73 ± 1.63^b^	0.002
C	14/14	9.86 ± 1.54^a^	15/15	22.73 ± 2.34^b^	<0.001
G	12/14	1.71 ± 0.35^a^	10/15	1.00 ± 0.22^a^	0.172
D	8/14	3.50 ± 1.37^a^	15/15	10.33 ± 1.89^b^	0.003
E1	8/14	3.79 ± 1.46^a^	15/15	11.60 ± 1.98^b^	0.002
E2	7/14	2.50 ± 0.94^a^	14/15	6.67 ± 1.17^b^	0.007
Sustained E2 > 10 min	5/14	0.71 ± 0.32^a^	12/15	1.73 ± 0.42^b^	0.031
**Total duration (min.)**					
Np	14/14	68.90 ± 19.24^a^	15/15	83.67 ± 10.87^a^	0.070
Probe	14/14	411.10 ± 19.24^a^	15/15	396.33 ± 10.87^a^	0.070
C	14/14	253.00 ± 21.00^a^	15/15	260.16 ± 14.14^a^	0.747
G	12/14	113.61 ± 23.82^a^	10/15	33.70 ± 7.41^b^	0.006
D	8/14	2.33 ± 0.88^a^	15/15	7.61 ± 1.54^b^	0.004
E1	8/14	3.53 ± 1.56^a^	15/15	16.37 ± 3.38^b^	0.001
E2	7/14	38.64 ± 18.66^a^	14/15	78.50 ± 20.03^b^	0.023
Sustained E2 > 10 min	5/14	32.82 ± 16.49^a^	12/15	64.57 ± 20.14^b^	0.039
**Mean duration (min.)**					
Np	14/14	16.72 ± 3.36^a^	15/15	9.93 ± 2.47^a^	0.133
Probe	14/14	153.12 ± 39.12^a^	15/15	74.85 ± 29.66^b^	0.002
C	14/14	34.48 ± 6.17^a^	15/15	13.79 ± 2.28^b^	<0.001
G	12/14	85.32 ± 18.85^a^	10/15	36.25 ± 5.68^b^	0.017
E1	8/14	0.95 ± 0.28^a^	15/15	1.38 ± 0.21^a^	0.155
E2	7/14	12.25 ± 4.42^a^	14/15	24.00 ± 13.85^a^	0.971
sustained E2 > 10 min	5/14	42.38 ± 15.29^a^	12/15	40.83 ± 15.52^a^	0.646
Mean duration of E2 per phloem phase	7/14	10.22 ± 4.35^a^	14/15	12.24 ± 12.24^a^	1.000
Duration of the 1st E2 in the recording	7/14	2.64 ± 1.24^a^	14/15	22.59 ± 13.36^b^	0.018
**Sequential variables**					
Time to first probe from start of EPG	14/14	21.91 ± 5.64^a^	15/15	13.82 ± 3.63^a^	0.234
Time from start of EPG to first sustained E2 > 10 min	5/14	386.65 ± 43.21^a^	12/15	283.33 ± 35.47^b^	0.049
Time to the 1st E2 from start of 1st probe	7/14	282.75 ± 53.53^a^	14/15	154.87 ± 35.69^a^	0.102
**Indices (% of time on a given waveform)**					
Percentage of probing spent in C	14/14	61.76 ± 4.29^a^	15/15	66.10 ± 3.68^a^	0.477
Percentage of probing spent in E1	8/14	0.804 ± 0.35^a^	15/15	4.098 ± 0.86^b^	<0.001
Percentage of probing spent in E2	7/14	8.39 ± 3.98^a^	14/15	19.02 ± 4.75^b^	0.016
Percentage of number of E2s that are sustained	5/14	21.80 ± 6.57^a^	12/15	31.63 ± 7.54^a^	0.524
Percentage of probing spent in G	12/14	28.52 ± 5.76^a^	10/15	8.84 ± 1.94^b^	<0.001

PPW, proportion of individuals that produced a specific waveform type period on lemon plants. Time is expressed in minutes. Means within a row followed by different letters are significantly different. Np, non‐probe; C, pathway; D, phloem contact; E1, phloem salivation; E2, phloem sap ingestion; G, xylem sap ingestion.

### 2.3 Open field mark–release–recapture assays

#### 2.3.1 Study area

Two lemon orchards, *C. limon* (L.) (variety ‘Lunario’) were selected, one located in Vale (Alvarelhos, Trofa, Porto district, 41° 18′ 03.3″ N, 8° 36′ 28.0″ W) and the other in Ribela (Vila Nova de Famalicão, Braga district, 41° 26′ 39.7″ N, 8° 30′ 15.7″ W). The first one was newly planted with 1‐year‐old plants (average height 70 cm) and the second had 2‐year‐old plants (average height 120 cm). Both study areas are currently located within the demarcated area of *T. erytreae* (DGAV, 2016–2021). According to the Köppen & Geiger classification, the region's climate is of the Csb type,^37^ characterized by warm summers of Mediterranean climate. The prevailing wind is ENE and W–E in Vale and Ribela, respectively.

#### 2.3.2 Description of orchards and experimental design

The orchard in Vale encompasses an area of 0.7 ha with flat topography, surrounded by corn crops and open grassland fields. The lemon plants were distributed in 14 rows in the orchard, interspaced by 3 m with 4 m between rows. A total of 90 plants were initially selected, and a series of three rows of quadrats were defined throughout the orchard according to the total number of plants per row of the plantation. The schematic design is presented in Fig. S2.

Each row of quadrats was separated by one row of the plantation. Each quadrat consisted of three rows of five plants following the plantation rows. Each row of quadrats encompassed a total of five, four and three quadrats, respectively. In turn, the three rows of quadrats encompassed three, two and one quadrat treated with kaolin, whereas the remaining quadrats were used as control (i.e. two control quadrats per row). The control quadrats were sprayed only with water. The central row of plants within each quadrat was initially planned to be inspected for marked individuals; however, as a consequence of the low number of recaptured individuals after the first time point (i.e. 8 h after the release, see below), all of the lemon plants within the orchard were inspected every time. The release of *T. erytreae* was made on 10 September 2020.

In Ribela, the selected orchard has an area of 0.4 ha and is part of a broader extension of citrus cultivation with a total area of 3.4 ha. It has a flat topography and plants are interspaced by 2 m arranged in a total of 16 rows with a between‐row distance of 6 m.

A grid of four rows encompassing 15 plants each (i.e. a total of 60 plants) was selected throughout the central area of the orchard. In each row, three blocks were sequentially delimited consisting of five plants each. The blocks were alternately defined as control or treated with kaolin (i.e. a total of six treated and six control blocks). Thus, rows 1 and 3 contained one control and two treated blocks each, whereas rows 2 and 4 contained two control blocks and one treated block each (Fig. S3).

Two assays were conducted in Ribela to assess the effect of the phenological state of the plants on the number of recaptured individuals. A first release was made on 8 July 2021 when the lemon plants were mostly flushing (plants with flush hereafter), and a second release on 22 July 2021 when the flushes were already mature (plants without flush hereafter).

#### 2.3.3 Application of commercial kaolin

Kaolin (Surround® WP, Tessenderlo Kerley Inc., Phoenix, AZ, USA) was applied using a standard 20‐L backpack mechanical sprayer. In order to ensure the kaolin effect, the reflected UVA light was measured at solar zenith in five plants (20 cm above the canopy) using an ALMEMO 25904S radiometer (Ahlborn GmbH, Holzkirchen, Germany) detecting the UV spectrum; experiments were conducted with a UVA light reflection of 4.17 ± 0.07 (mean ± SE) in treated plants and 0.78 ± 0.03 (mean ± SE) in control plants. In Vale, the first application of kaolin (3%) was made on 18 July 2020 and subsequent applications of kaolin (2%) were made on a biweekly basis until 9 September 2020. In each application, the kaolin was sprayed on the 15 plants of the corresponding quadrat. In Ribela, a total of three applications of kaolin were done on 2, 8 and 22 July 2021, respectively.

#### 2.3.4 *Origin, preparation and method of release of adult individuals of* T. erytreae

A total of three mark–release–recapture experiments were conducted in two orchards (see Section 2.3.1.), the first one in Vale in 2020; however, owing to the low number of recaptured individuals, a second experiment (encompassing two assays, one in plants with flush and one in plants without flush) was conducted in Ribela.

In Vale, the adults of *T. erytreae* were obtained in the pedagogical orchard ‘Quinta do Crasto’. A total of 2000 individuals were captured on lemon and tangerine trees and placed in eight Falcon tubes (50 mL) (250 individuals per tube). Once the individuals were distributed among the tubes, a small amount of fluorescent blue dust (Day‐Glo Color Corp. Cleveland, OH, USA) was deposited into each Falcon tube (3 mg per tube). Each tube then was carefully rotated by hand for 30 s to sprinkle the marker on the insects. Once marked, the individuals were placed in eight lemon seedlings (i.e. 250 individuals per plant) for 48 h to feed. On the release day (9 September 2020), the seedlings were transported to the field and placed at ground level at eight release points (i.e. 250 marked individuals per point). The release points were located 8 m away from the border rows of the plantation and distributed along a single row so that the northernmost and southernmost row encompassed five and three release points, respectively. At the moment of release (10:00 hours), each seedling was cut and fixed horizontally on a plastic support (12.5 cm high) to avoid the contact with the soil heat and allow the marked individuals to fly.

The individuals used for the releases conducted in Ribela in 2021 also were obtained from the orchard ‘Quinta do Crasto’, In this case, the individuals were distributed among four Falcon tubes (500 mL) with 500 individuals per tube (i.e. a total of 2000 individuals each release), marked as depicted before, and immediately transported in the tubes to the field to be released. Four releasing points were established within the grid of plants, two points between the first and second row of plantation and two points between the third and fourth row of plantation coinciding with the change of blocks. At each releasing point, a fine wood stick (1 m high, 7 mm diameter) was pushed vertically into the ground, and a Falcon tube was fixed vertically at the tip of the stick with the opening facing up. The four tubes were opened at 10:00 hours, allowing individuals to fly.

The evaluation of the recapture of individuals was made by direct visual search of marked insects on the plants. In each tree, all the branches (*i.e*., flushing or not) were inspected. In Vale, the observations were made 8, 24, 72, and 120 h after the release, whereas in Ribela, observations occurred 6, 12, 24, and 48 h after release.

### 2.4 Data analysis

#### 2.4.1 Dual‐choice test in laboratory

The effect of kaolin application on the plant selection of *T. erytreae* was assessed using a generalized linear model (GLM) with Poisson distribution, developed with the number of individuals counted at each time point as a response variable and sex, time point and treatment (control plant, kaolin plant and cage) were explanatory variables. A first full model was developed, including an interaction term for treatment and time lapse. Variable selection was conducted toward an optimal model using backward selection following Zuur *et al*.^38^ Finally, a Tukey *post hoc* test (α = 0.05) was used to compare factor levels for treatment. The GLMs were developed using R.^39^

#### 2.4.2 *Effect of kaolin on the probing and feeding behavior of* T. erytreae

EPG variables were processed for each given insect with the help of an excel Data Workbook CSIC‐UAL elaborated by Garzo E. (Institute of Agricultural Sciences, CSIC, Spain) and Alvarez A.J. (University of Almería, Spain). Data were obtained for each of the EPG variables and the two treatments (kaolin‐treated and untreated plants). First, a series of transformations of the data obtained were carried out using the Ln (× + 1) transformation. Then, a Shapiro–Wilk test was used to check for normality and a Bartlett test to check for the homoscedasticity of the data. The results of these tests indicated that normality or homoscedasticity of the variances was not fulfilled. Therefore, a nonparametric Mann–Whitney *U*‐test (α = 0.05) was used to compare each of the EPG variables between the two treatments. The proportion of individuals that produced a specific waveform type (PPW) was compared between treatments using a χ^2^ test followed by the Fisher's Exact test if the expected values were <5. This analysis was conducted using Spss and statview 4.0 software (Abacus Concepts, Berkeley, CA, USA).

#### 2.4.3 *Effect of kaolin on the settling behavior of* T. erytreae *in the field*

The number (*N*) of recaptured individuals was counted in each plant at each time point after release. Due to the low number of recaptures in Vale no further data analysis was conducted for this orchard. For Ribela, a Welch *t*‐test for unequal variances was used to compare the number of recaptured individuals in plants with and without flush.

The effect of the application of kaolin on the landing and settlement of *T. erytreae* was assessed using generalized estimating equations (GEEs). This method is an extension of the GLM used to analyze low replicated count data as an alternative to more complex generalized mixed models.^38^, ^40^ GEEs can be used to estimate the within‐subject correlation (ρ). If this correlation is found to be low, then GLMs can be used for modelling purposes regardless of replication.^30^ An interchangeable correlation structure was assumed (i.e. a single correlation parameter, ρ). The six blocks were used as replicates (random term), whereas treatment (kaolin *versus* control), and time point were used as explanatory variables with Poisson‐like distribution and logarithmic link function. Because the correlation parameter was low (ρ = 0.003), GLMs with negative binomial distribution to deal with potential overdispersed data were used hereafter so that two models were developed, one for plants with flush and one for plants without flush. A *post hoc* analysis was conducted for each model to compare the different time points. Finally, for each assay and time point, Welch *t*‐tests for unequal variances were used to compare the number of recaptured individuals between treated and control plants. The GLMs were developed using R.^39^

## 3 RESULTS

### 3.1 Dual‐choice test in laboratory

**Figure 1:**
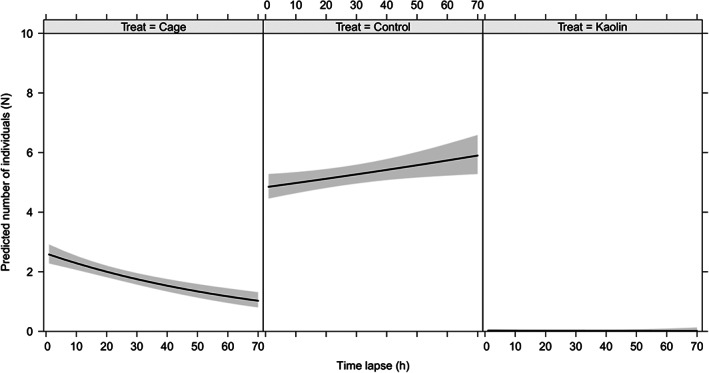
Number of living individuals found on the cage (insects walking around the cage, on kaolin‐treated and on untreated control plants over time. Gray areas represent 95% confidence interval.

The number of individuals settled on control untreated plants during the dual‐choice assay was significantly higher than the number of individuals settled on kaolin‐treated plants and those walking or standing on the cage (χ^2^ = 2582.45; df = 2; *P* < 0.001) (Fig. 1). As expected, the higher the number of insects found settled on plants, the lower the number of insects wandering on the cage over time; most of the psyllids released preferred to settle on untreated plants after 72 h (5.60 ± 0.32 males; 5.80 ± 0.35 females) than on kaolin‐treated plants (0.00 ± 0.00 males; 0.03 ± 0.03 females) (mean ± SE).

However, the variable time did not significantly affect the choice for settling (χ^2^ = 1.85; df = 1; *P* = 0.173) and the sex of individuals did not significantly influence settling behavior (χ^2^ = 0.42; df = 1; *P* = 0.517) (Fig. 1). After model selection, the treatment (χ^2^ = 2582.45; df = 2; *P* < 0.001) and the interaction term for treatment and time were found to be significant (χ^2^ = 77.56; df = 2; *P* < 0.001) (Fig. 1).

### 3.2 Effect of kaolin on the probing and feeding behavior of *T. erytreae*

**Figure 2:**
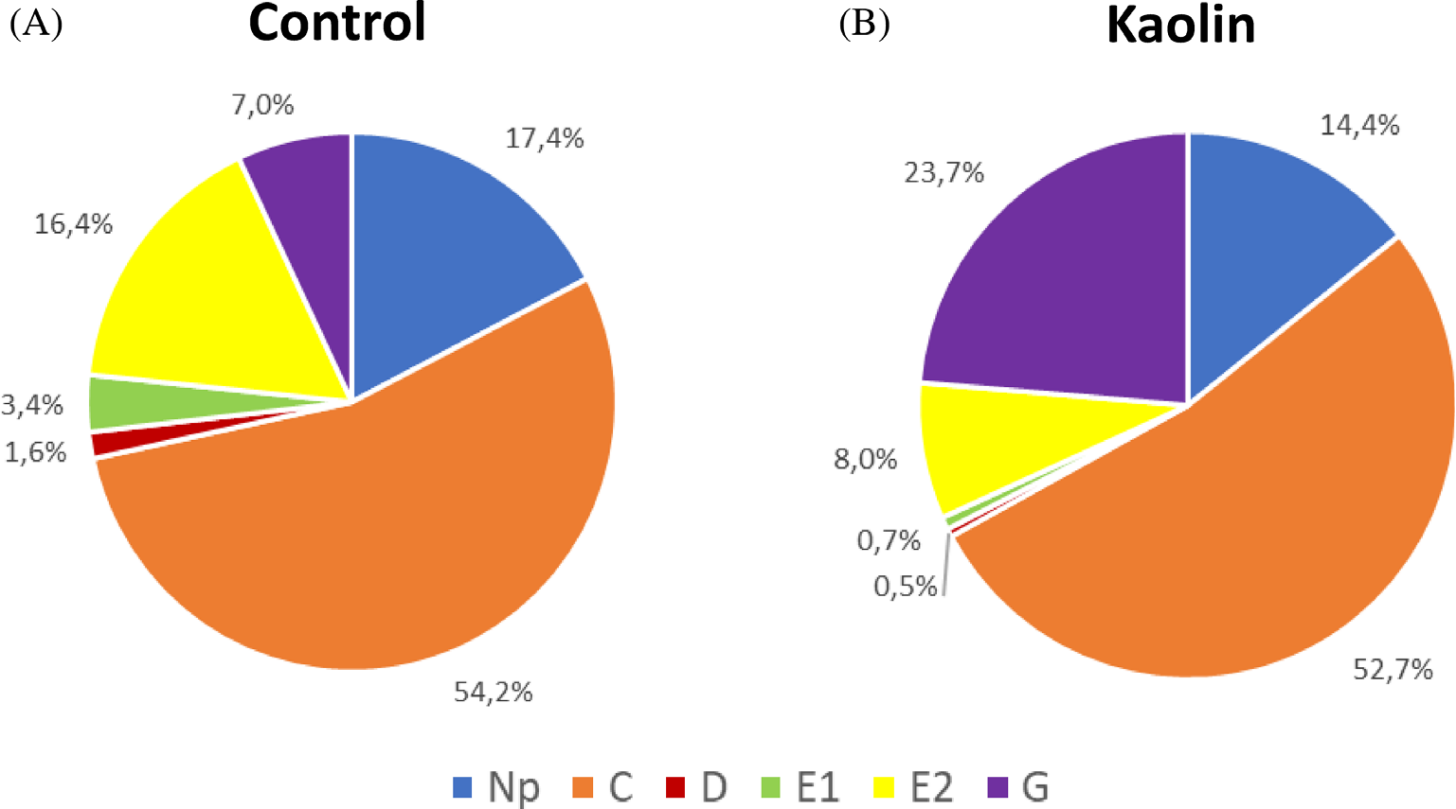
Percentage of the total duration of each waveform event (Waveform: Np, non‐probe; C, pathway; D, phloem contact; E1, phloem salivation; E2, phloem sap ingestion, and G, xylem sap ingestion) spent by *T. erytreae* on kaolin‐treated and untreated lemon plants.

**Figure 3:**
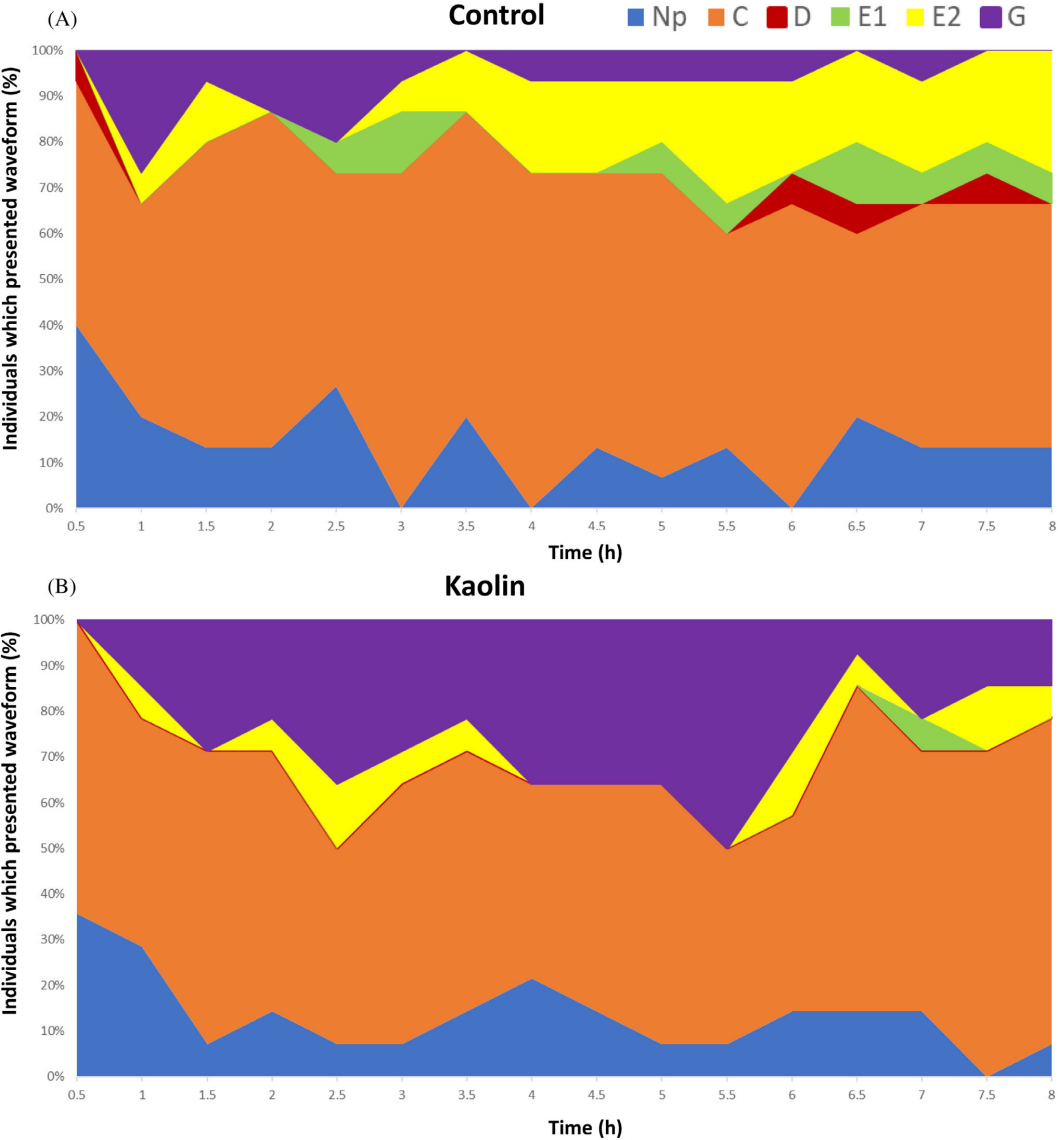
Percentage of individuals of *T. erytreae* exhibiting specific waveforms at 30‐min intervals over a total recording time of 8 h on kaolin‐treated and untreated lemon plants.

The overall results show that *T. erytreae* prefers to feed on the untreated control than on kaolin‐treated plants. The percentage of the total time spent on each phloem phase activities (waveforms D, E1 and E2) by the psyllid cohort over time increased on control plants compared to kaolin‐treated plants (Fig. 2). The main difference observed between treatments was the percentage of time spent in phloem and xylem ingestion. When exposed to kaolin‐treated plants, psyllids spent more extended periods on xylem ingestion and less time on phloem ingestion (Fig. 2). A higher percentage of individuals remained in phloem ingestion (E2) on the control than on the kaolin‐treated plants, mainly after the 3rd hour of exposure (Fig. 3: control 6.67%–26.67%; kaolin 7.14%–14.29%). However, a high percentage of individuals exposed to kaolin‐treated plants spent their time on xylem ingestion activities throughout the recording period compared to the control plants (Fig. 3: control 0%–26.67%; kaolin 0%–50%).

Thirty‐three EPG variables were considered to analyze the probing and feeding behavior of *T. erytreae* when exposed to kaolin‐treated plants (Table 1).

No significant differences were observed in the total duration of no‐probes (Np) and probes between treatments, but the number and mean duration of probes and no‐probes were significantly different. Psyllids exhibited significantly fewer nonprobing and probing events on kaolin‐treated than control plants (Table 1). However, the mean duration of the probes was significantly longer on plants treated with kaolin (153.12 ± 39 min) than on control plants (74.85 ± 29.66 min) (U = 37, *P* = 0.002). Likewsie, the mean duration of time that psyllids spent in intercellular stylet pathway (waveform C) was significantly longer on kaolin‐treated plants (34.48 ± 6.17 min) than on control plants (13.79 ± 2.28 min) (*P* < 0.001). The EPG results did not show evidence of delay in the initialization of the first probe when psyllids were placed on kaolin‐treated plants. In both kaolin‐treated and untreated plants, the psyllids started probing soon after the start of the recording and with no significant differences between treatments (time to first probe from the start of EPG: 21.91 ± 5.64 and 13.82 ± 3.63 min, respectively, *P* = 0.234).

The number and total duration of the phloem activities (D, E1, E2 and sE2) was clearly affected by the kaolin treatment. *T. erytreae* exhibited significantly fewer and shorter duration phloem‐related events on kaolin‐treated than untreated plants (Table 1). Also, the psyllid spent less time in phloem sap ingestion (duration of the 1st E2 in the recording and percentage of probing spent in E2) on the kaolin‐treated than on the control plants (Table 1). Furthermore, kaolin delayed sustained phloem sap ingestion by *T. erytreae*, as shown by the time spent from the start of EPG to first sustained phloem ingestion (E2 > 10 min) which was significantly longer on plants treated with kaolin (386.65 ± 43.21 min) than on the untreated control (283.33 ± 35.47 min) (*P* = 0.049).

However, no significant differences were found in the mean duration of phloem activities between kaolin‐treated and control plants (Table 1). Nevertheless, a significantly lower proportion of individuals was able to engage in phloem ingestion activities when exposed to kaolin than on control plants. In fact, only half of the individuals (PPW seven of 14) exposed to kaolin‐treated plants were able to ingest the phloem whereas almost all were able to engage in phloem sap ingestion when exposed to untreated control plants (PPW 14 of 15) (χ^2^ = 6.807; *P* = 0.014). Moreover, only five of 14 individuals exposed to kaolin‐treated plants were able to show a sustained phloem sap ingestion compared to 12 of 15 individuals on the untreated control (χ^2^ = 5.854; *P* = 0.025). Furthermore, no significant differences between treatments were found in the number of psyllids that showed xylem ingestion events (Table 1: kaolin 1.71 ± 0.35; control 1.00 ± 0.22; *P* = 0.172) (PPW 10 of 15 control, 12 of 14 kaolin; χ^2^ = 1.435; *P* = 0.390). However, the total duration and mean duration of time that psyllids spent on xylem ingestion were significantly higher (more than twice those exposed to the untreated control). Also, the percentage of probing time spent in xylem sap ingestion was significantly higher on kaolin‐treated than on control plants (kaolin 28.52 ± 5.76; control 8.84 ± 1.94; *P* < 0.001) (Table 1).

### 3.3 Open field mark–release–recapture assays

**Figure 4:**
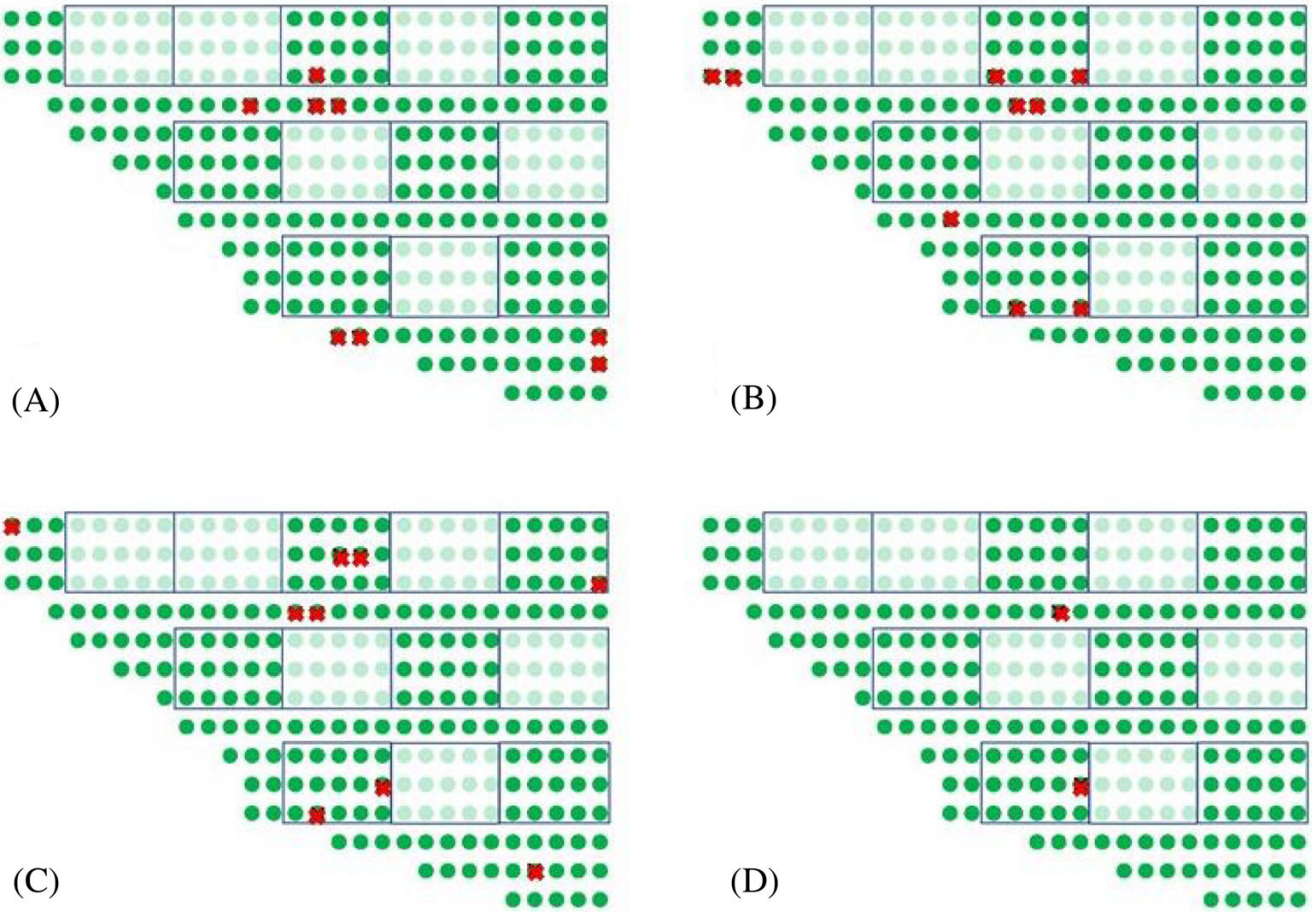
(A) Position where *T. erytreae* individuals were recaptured within the orchard 8 h (A1), 24 h (A2), 72 h (A3) and 120 h (A4) after release in Vale. Squares, initially selected quadrats of plants; green dots, untreated (control) plants; light green dots, sprayed (kaolin) plants. Crosses indicate the plants where at least one individual was found at each time lapse after release. (B) Schematic view of the sampling design conducted in Vale.

A total of 40 marked individuals were recaptured at 23 control plants at the end of the assay in Vale in 2020. After the 8, 24, 72 and 120 h time points, 14, 14, nine and three individuals were recaptured in eight, nine, eight, and two control plants, respectively. No individuals were found on treated plants (Fig. 4).

**Figure 5:**
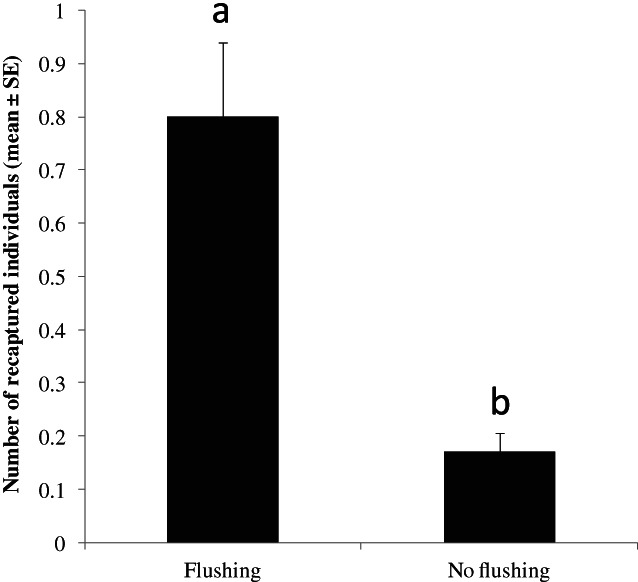
Marked individuals of *T. erytreae* recaptured on lemon plants at different phenological states (plants with and without flush) in open field in 2021 (Ribela, Portugal). Different letters above bars indicate a significantly different number of individuals recaptured for each flushing status (α = 0.001).

In 2021 in Ribela, 192 individuals were recaptured during the first assay (plants with flush), whereas 41 individuals were recaptured during the subsequent assay (plants without flush). The mean number of recaptured individuals per plant was significantly higher (*t* = 4.37, *P* < 0.001) in the assay with plants with flush compared with plants without flush (Fig. 5). During the assay with flush, 92, 50, 30 and 20 individuals were recaptured after 6, 12, 24 and 48 h, respectively. However, 12, 12, 12 and five individuals were recaptured after 6, 12, 24 and 48 h, respectively, during the assay without flush.

**Figure 6:**
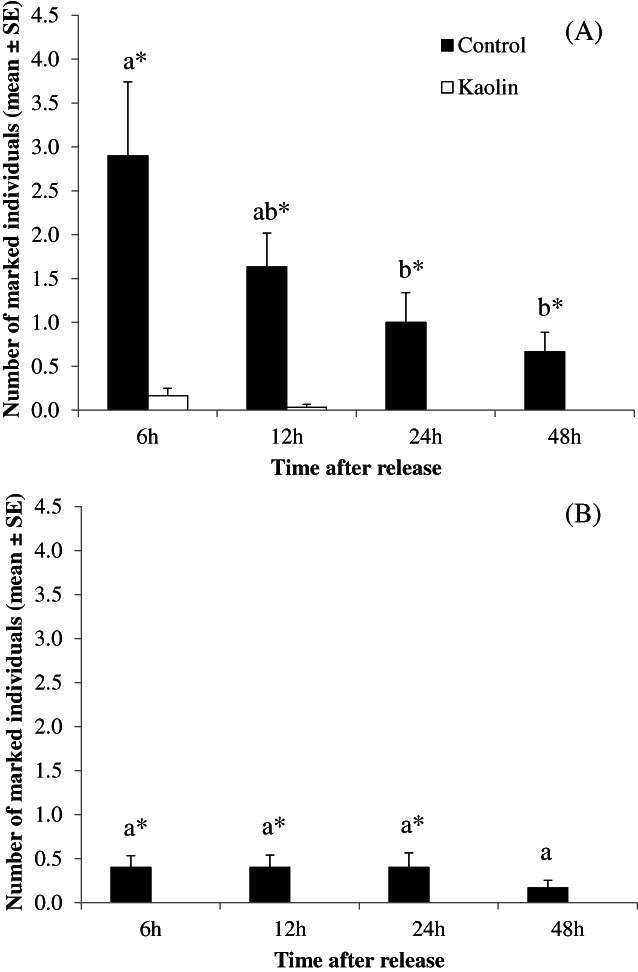
Number of marked individuals of *T. erytreae* recaptured after release on lemon plants at different time points and phenological states in open field (Ribela, Portugal). (A) Plants with flush (8 July 2021); (B) plants without flush (22 July 2021) (no individuals were found in treated plants in this assay). Different letters above bars indicate a significantly different number of individuals recaptured for each time point (α = 0.001). Asterisks above bars indicate significant differences between treatments for each time point (α = 0.05).

The number of individuals recaptured in control plants was significantly higher compared with treated plants at each time point in the experiment with plants with flush (χ^2^ = 113.0, df = 1, *P* < 0.001) [Fig. 6(A)]. The same pattern was found for plants without flush except 48 h after release (χ^2^ = 47.6, df = 1, *P* < 0.001) [Fig. 6(B)]. The number of recaptured individuals significantly decreased with time during the assay with flush (χ^2^ = 23.0, df = 3, *P* < 0.001) [Fig. 6(A)], whereas no significant differences were found during the assay without flush (χ^2^ = 2.9, df = 3, *P* < 0.41) [Fig. 6(B)].

## 4 DISCUSSION

In this work, the spray of kaolin clay significantly influenced the settling and feeding behavior of *T. erytreae* adults in the laboratory and had a deterrent effect on its settling behavior on lemon plants in the field. The inhibition of the settling behavior of *T. erytreae* during the dual‐choice assay in the laboratory agrees with the results observed for *D. citri* by Miranda *et al*.^4^ in laboratory no‐choice assays. Miranda *et al*.^4^ observed a reduction of ≤40% in adult insects settled on plants treated with 3%, 5% and 7% kaolin solutions. Color is an important factor for host location by psyllids such as *T. erytreae* and *D. citri*.^41^ Urban^42^ stated that *T. erytreae* adults are positively phototactic for light wavelengths ≈550 nm, representing the luminous frequency between yellow and green. Thus, the preference of the insects for the control plants in the laboratory could be mediated by the visual camouflage effect resulting from the total coverage of the plants with the kaolin solution.

In the open‐field experiments, no individuals were found on kaolin‐treated plants in Vale; however, the total number of recaptured individuals was extremely low (2.00%). In Ribela, 9.60% of individuals were recaptured after the assay in plants with flush, whereas 2.05% of individuals were recaptured in plants without flush. These results agree, in general, with Tomaseto *et al*.^43^ who found a significantly higher amount of fluorescent‐marked individuals of the other vector of HLB, *D. citri*, in plants with the presence of young citrus leaves in São Paulo (Brazil). Also, Lewis‐Rosenblum *et al*.,^44^ using an *in situ* protein marking technique, found that the marked psyllids captured increased with the density of emerging young leaves of sweet orange in Florida (USA). In this study, the trees were inspected before the second release of marked individuals in Ribela to check for settled individuals corresponding to the first release (i.e. during tree flushing). Because no individuals were found, we assumed that all of the newly found marked individuals corresponded to the second release (i.e. trees with no flushing). We recommend considering this in further studies involving sequential releases to avoid re‐counting individuals, which could lead to potential statistical biases.

The difference between the number of individuals recaptured in plants with and without flush in Ribela can be explained in terms of *T. erytreae* bioecology. Females of *T. erytreae* show a strong preference for plants with flush because the oviposition is restricted to tender shoots.^45^ In fact, Martini *et al*.^46^ found that young emerging leaves were the major factor driving the population of *D. citri* during winter in commercial groves in Florida when the number of shoots is extremely reduced, and the available sites for oviposition significantly decrease.

The low number of recaptured individuals in Vale also could result from this behavior in *T. erytreae*. Moreover, the citrus grove in Vale is unprotected from wind and point measures of wind velocity reached up to 14.40 km h^−1^. According to Martini *et al*.,^47^ windbreaks significantly influence the spread pattern of *D. citri*. Moreover, although wind direction may not be correlated with the number of marked psyllids recaptured,^24^ psyllids, including *Trioza* spp., show remarkable migration capabilities travelling distances of several kilometers.^48^

Regarding the application of kaolin, the number of recaptured individuals in this work was always significantly higher in control plants. Indeed, individuals that landed on kaolin‐sprayed plants were found only in plants with flush and during the first 12 h. This suggests that the clay particles may have a deterrent effect on *T. erytreae* regarding the selection of the landing site and the site tenacity. These results agree with those obtained by Miranda *et al*.^22^ in Araraquara (Brazil), who recorded a significantly lower number of *D. citri* on plants sprayed with kaolin every 7 and 14 days in comparison to the control plants. Ramírez‐Godoy *et al*.^49^ in Apulo (Colombia) observed a reduction in the adult, nymph and egg populations of *D. citri* as a consequence of foliar applications of kaolin compared to untreated plants. In Florida, Pierre *et al*.^50^ found lower numbers of individuals of *D. citri* in sweet orange plants treated with red and white kaolin than in plants in which foliar insecticide was applied. However, Hall *et al*.^21^ observed less adult movement, settling and oviposition on kaolin‐treated citrus trees than on control trees.

The effectiveness of kaolin is related to mechanically derived aspects such as more difficult attachment and probing^4^, ^51^ and wavelength‐dependent interactions such as interference on visual cues.^52^ In our study we evaluated the feeding behavior on young leaves of kaolin‐treated and untreated lemon seedlings, and we observed that the total duration of the xylem ingestion was 33‐fold higher on kaolin‐treated than on untreated plants. Our results could be correlated with those of Pompon *et al* ^53^ who observed a positive direct relationship between the level of dehydration of aphids after a period of starvation and xylem ingestion. The reduction of time spent in phloem sap ingestion by *T. erytreae* on treated plants also suggests that processed kaolin could be an efficient tool to reduce HLB transmission in lemon orchards.

## 5 CONCLUSION

The application of kaolin clay was helpful to prevent the landing, settling behavior and feeding of *T. erytreae* on lemon plants in the laboratory and in the field. Kaolin is an efficient and widely used substance for sustainable management as an alternative to synthetic insecticides and can be implemented in the citrus agroecosystem to reduce or prevent the landing of *T. erytreae*. This is especially important during plant flushing because, during this period, citrus plants are susceptible to the attack of *T. erytreae*. Also, kaolin could effectively prevent *T. erytreae* landing, especially on young citrus plants such as the ones we used for our open‐field experiments. Moreover, even if the adults were able to settle, the kaolin clay film had a deterrent effect on the feeding of *T. erytreae*, thus reducing the risk of transmission of HLB. However, further research must be conducted on the most efficient dose and best timing to spray, the mode of application (e.g. different models of a backpack sprayer, tractor application), the effect of different kaolin types (e.g. red kaolin), the frequency and season of applications and the potential adverse effects on natural enemies. Notwithstanding this, kaolin clay represents a promising tool for limiting or preventing the spread of the HLB disease throughout Europe.

## CONFLICT OF INTEREST

The authors declare no conflict of interest.

## Supporting information


**Figure S1.** Example of one cage containing a control and a kaolin‐treated plant. The release platform for the individuals of *Trioza erytreae* can be observed between the two plants.
**Figure S2.** Schematic experimental design of the assay conducted in Vale (Trofa).
**Figure S3.** Schematic experimental design of the assay conducted in Ribela (Vila Nova de Famalicão).

## Data Availability

The data that support the findings of this study are available from the corresponding author upon reasonable request.
