# Supplementary material for: Kaolin particle films disrupt landing, settling behavior and feeding of Trioza erytrae on lemon plants
Source: Pest Manag Sci. 2022 Aug 11;78(11):4753–63. doi: 10.1002/ps.7095 (PMC9805282; doi:10.1002/ps.7095)
Supplement: Supplementary file 1 — Figure S1. Example of one cage containing a control and a kaolin‐treated plant. The release platform for the individuals of Trioza erytreae can be observed between the two plants. Figure S2. Schematic experimental design of the assay conducted in Vale (Trofa). Figure S3. Schematic experimental design of the assay conducted in Ribela (Vila Nova de Famalicão). [file PS-78-4753-s001.docx]

**Supplementary material**


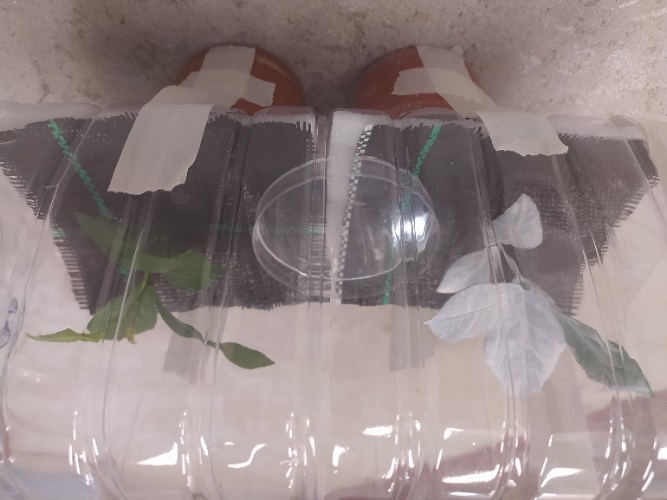


**Figure S1**. Example of one cage containing a control and a kaolin-treated plant. The release platform for the individuals of *Trioza erytreae* can be observed between the two plants.


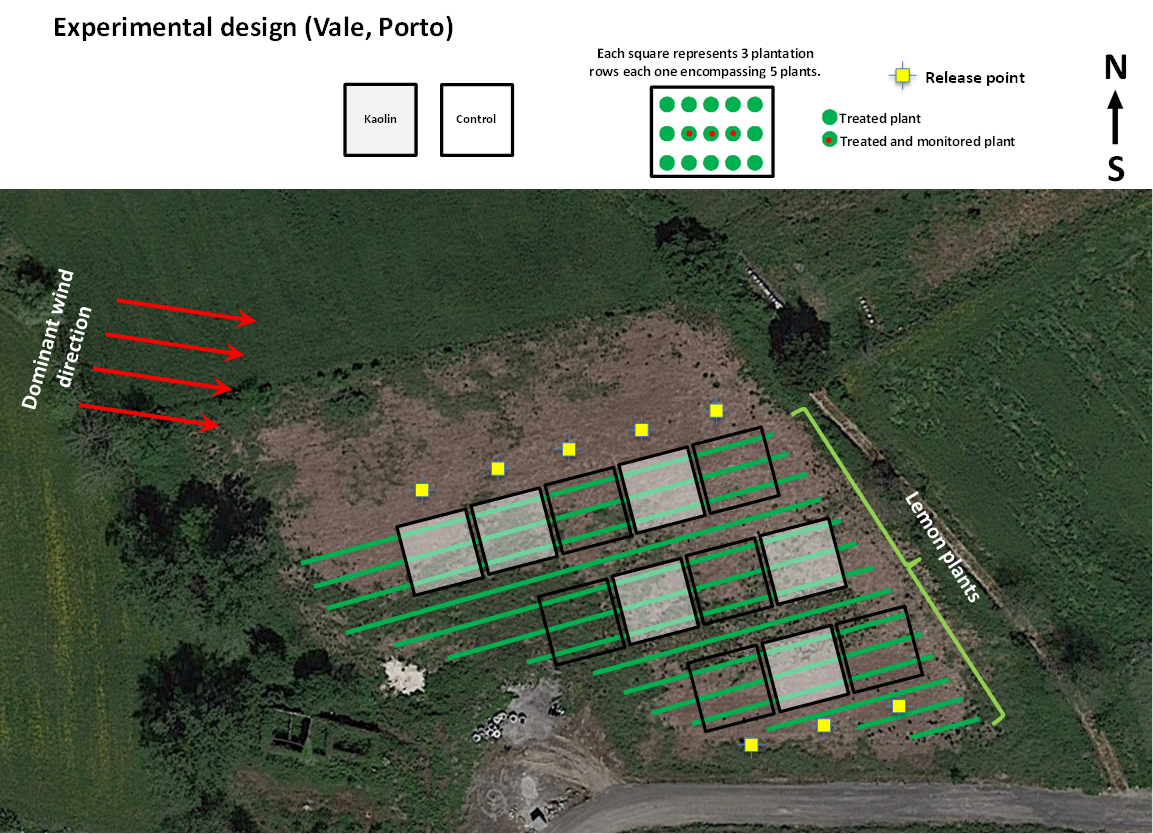


**Figure S2.** Schematic experimental design of the assay conducted in Vale (Trofa).

**Figure S3.** Schematic experimental design of the assay conducted in Ribela (Vila Nova de Famalicão).
